# Importance of E3 ubiquitin ligase Synoviolin in fibrogenesis

**DOI:** 10.1186/ar3676

**Published:** 2012-02-09

**Authors:** Naoko Yagishita, Daisuke Hasegawa, Satoko Aratani, Yoshihisa Yamano, Toshihiro Nakajima

**Affiliations:** 1 Institute of Medical Science, St.Marianna University School of Medicine, Kawasaki, Kanagawa 216-8512 Japan; 2 Institute of Medical Science, Tokyo Medical University, Shinjuku-ku, Tokyo 160-8402 Japan; 3 Bayside Misato Medical Center, Kochi 781-0112 Japan

## 

The symptoms of rheumatoid arthritis (RA) are based on the many processes; chronic inflammation, overgrowth of synovial cells, bone and joint destruction and fibrosis. To clarify the mechanism of outgrowth of synovial cells, we carried out immunoscreening using anti-rheumatoid synovial cell antibody, and cloned 'Synoviolin'. Synoviolin, a mammalian homolog of Hrd1p/Der3p, is endoplasmic reticulum (ER)-resident E3 ubiquitin ligases with a RING motif, and is involved in ER-associated degradation (ERAD). Synoviolin is highly expressed in synoviocytes of patients with RA. Overexpression of synoviolin in transgenic mice leads to advanced arthropathy caused by reduced apoptosis of synoviocytes. We postulate that the hyperactivation of the ERAD pathway by overexpression of synoviolin results in prevention of ER-stress-induced apoptosis leading to synovial hyperplasia. Indeed, synoviolin^+/- ^knockout mice showed resistance to the development of collagen-induced arthritis owing to enhanced apoptosis of synovial cells. In addition, Synoviolin ubiquitinates and sequesters the tumor suppressor p53 in the cytoplasm, thereby negatively regulating its biological functions in transcription, cell-cycle regulation and apoptosis by targeting it for proteasomal degradation. Therefore Synoviolin regulates, not only apoptosis in response to ER stress, but also a p53-dependent apoptotic pathway. These studies indicate that Synoviolin is one of the causative factors of arthropathy. Further analysis using gene targeting approaches showed that in addition to its role in RA, Synoviolin is essential for embryogenesis. Synoviolin deficient (*syno*^-/-^) mice exhibited severe anemia caused by enhancement of apoptosis in fetal liver, and the results suggested that the liver is sensitive organ for Synoviolin.

Thus, this study aimed to explore the involvement of the Synoviolin in fibrosis process of RA using mice model of liver fibrosis. In CCl_4_-induced hepatic injury model, *syno*^+/- ^mice are resistant to onset of liver fibrosis. The number of activated HSCs was decreased in *syno*^+/- ^mice, and some of these cells showed apoptosis. Furthermore, collagen expression in HSCs was upregulated by synoviolin overexpression, while synoviolin knockdown led to reduced collagen expression. Moreover, in *syno*^-/- ^MEFs, the amounts of intracellular and secreted mature collagen were significantly decreased, and procollagen was abnormally accumulated in the endoplasmic reticulum. In conclusion, Synoviolin is involved in not only overgrowth process of synovial cells but also fibrosis process.

